# Inflammatory Biomarkers as Differential Predictors of Antidepressant Response

**DOI:** 10.3390/ijms16047796

**Published:** 2015-04-08

**Authors:** Kenji Hashimoto

**Affiliations:** Division of Clinical Neuroscience, Chiba University Center for Forensic Mental Health, 1-8-1 Inohana, Chiba 260-7680, Japan; E-Mail: hashimoto@faculty.chiba-u.jp; Tel.: +81-43-226-2517; Fax: +81-43-226-2561

**Keywords:** antidepressant, biomarker, cytokine, inflammation, ketamine, predictor, response

## Abstract

Although antidepressants are generally effective in the treatment of major depressive disorder (MDD), it can still take weeks before patients feel the full antidepressant effects. Despite the efficacy of standard treatments, approximately two-thirds of patients with MDD fail to respond to pharmacotherapy. Therefore, the identification of blood biomarkers that can predict the treatment response to antidepressants would be highly useful in order to improve this situation. This article discusses inflammatory molecules as predictive biomarkers for antidepressant responses to several classes of antidepressants, including the *N*-methyl-d-aspartate (NMDA) receptor antagonist ketamine.

## Introduction

Major depressive disorder (MDD) is the most prevalent psychiatric disorder, and is also amongst the most severe and debilitating. The World Health Organization estimates that more than 350 million individuals of all ages suffer from depression [1]. Although antidepressants are generally effective in the treatment of MDD, it can still take weeks before patients feel the full antidepressant effects. Despite the efficacy of standard treatments, approximately two-thirds of patients with MDD fail to respond to pharmacotherapy. Furthermore, there is a high rate of relapse, and MDD patients have a high risk of attempting suicide. Therefore, the identification of biomarkers that can predict the treatment response to antidepressants would be very useful to improve this situation [2,3,4]. 

Accumulating evidence suggests that inflammatory processes play a role in the pathophysiology of MDD [5,6,7]. When bacterial endotoxin lipopolysaccharide (LPS) is administered to rodents, depression-like behaviors are induced after the induction of inflammation [8,9,10,11]. Furthermore, antidepressants, including selective serotonin reuptake inhibitors (SSRIs) and serotonin and norepinephrine reuptake inhibitors (SNRIs), could prevent depression-like behavior and alternations in serum pro-inflammatory cytokines, such as tumor necrosis factor-α (TNF-α), caused by LPS administration [12]. A meta-analysis showed the higher serum TNF-α levels in drug-free MDD patients compared with those in healthy controls [13]. A study using postmortem brain samples showed elevated gene expression of pro-inflammatory cytokines in the frontal cortex of people with a history of MDD [14]. Taken together, it is likely that both peripheral and central inflammation are associated with MDD and that anti-inflammatory drugs could ameliorate depressive symptoms in MDD patients.

C-reactive protein (CRP) is an annular ring-shaped, pentameric protein found in the blood plasma, and CRP levels were increased in response to inflammation. Meta-analyses showed that MDD is associated with increased CRP levels [15,16]. Recently, Uher *et al.* [17] reported an inflammatory biomarker as a differential predictor of clinical outcome in depression therapy. In a multicenter open-label randomized trial, the authors measured CRP, a commonly detectable biomarker of systemic inflammation, in patients with MDD who were randomly allocated to either 12 weeks of treatment with escitalopram (a SSRI, *n* = 115) or nortriptyline (a norepinephrine (NE) reuptake inhibitor (NRI), *n* = 126). Serum CRP levels at baseline differentially predicted treatment outcomes with both antidepressants. Patients with low levels of CRP (<1 mg/L) showed improvement on the Montegomery-Åsperg Depression Rating Scale (MADRS), with scores three points higher after escitalopram treatment, compared with nortriptyline. In contrast, patients with higher CRP levels scored three points higher on MADRS after nortriptyline, compared with escitalopram [17]. 

**Figure 1:**
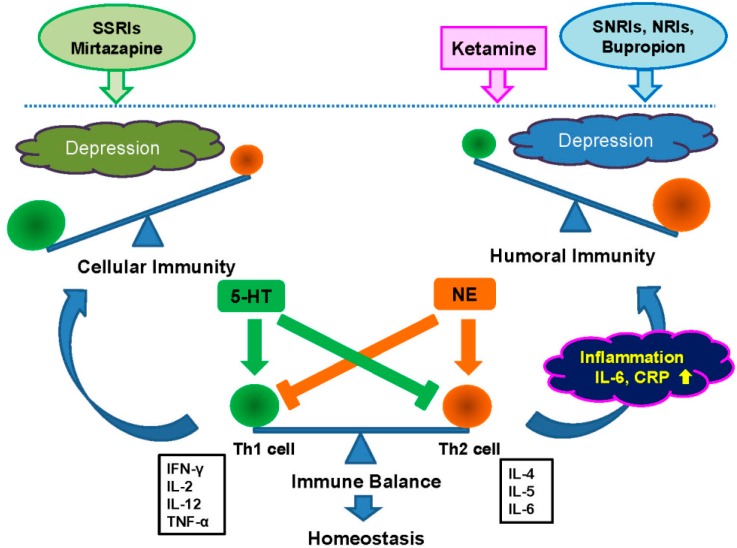
The balance between Th1 (cellular) and Th2 (humoral) response to the adaptive immune system. The immune system, composed of Th1-mediated cellular immunity and Th2-mediated humoral immunity, is essential to maintain health. Both Th1 and Th2 immunity are tightly controlled, but abnormalaties of the “immune balance” between Th1 and Th2 immunity is implicated in the pathophysiology of MDD. Th2 shift causes inflammation and increase in CRP protein and pro-inflammatory cytokines (e.g., IL-6), resulting in depressive symptom. Th1 shift also causes depressive symptom. 5-HT and NE are known to confer differential effects on inflammation. 5-HT and NE mediate a Th1 shift and a Th2 shift, respectively. Furthermore, 5-HT inhibits the production of Th2 cytokines such as IL-6, whereas NE inhibits production of Th1 pro-inflammatory cytokines, including TNF-α [18]. The SSRIs (e.g., paroxetine, sertraline, fluoxetine, escitalopram) cause a Th1 shift. The SNRIs (venlafaxine, duloxetine), and NRIs (nortriptyline, reboxetine) cause a Th2 shift [18]. Bupropion and mirtazapine may induce Th2 and Th1 shift, respectively [18]. In addition, the NMDA receptor antagonist ketamine may cause a Th2 shift. Thus, the regulation of the “immune balance” between Th1 and Th2 immunity is critical for therapy of MDD.

NE and 5-HT are known to confer differential effects on inflammation, and mediate a T helper 1 (Th1) shift and a T helper 2 (Th2) shift, respectively. Furthermore, 5-HT inhibits the production of Th2 cytokines such as interleukin 6 (IL-6), whereas NE inhibits production of Th1 pro-inflammatory cytokines, including tumor necrosis factor-α (TNF-α) (Figure 1) [18]. The SSRIs (e.g., paroxetine, sertraline, fluoxetine, escitalopram) cause a Th1 shift. The 5-HT and NE reuptake inhibitors (SNRIs; venlafaxine, duloxetine), and NRIs (reboxetine) cause a Th2 shift [18]. Furthermore, bupropion (a NE and dopamine reuptake inhibitor) and mirtazapine (NaSSA: NE and Specific Serotonergic Antidepressant) may induce Th2 and Th1 shift, respectively. Thus, the antidepressants that affect 5-HT and NE distinctly affect immunity: while NRIs suppress Th1-type cytokines and shift the balance toward humoral immunity. The SSRIs reduce the production of Th2-type cytokines and shift the balance toward cellular immune response (Figure 1) [17,18]. It would therefore be of great interest to examine whether serum levels of IL-6 and TNF-α could serve as reliable biomarkers for a clinical response to these two antidepressants (escitalopram and nortriptyline) in this cohort sample.

The *N*-methyl-d-aspartate receptor antagonist, ketamine, is the most attractive antidepressant therapy for patients with treatment-resistant MDD [19,20,21,22,23,24,25,26,27,28]. A single subanesthetic dose (0.5 mg/kg) of ketamine produces a rapid antidepressant effect in two-thirds of these treatment resistant MDD patients, which can last for over a week [20,21]. However, biomarkers able to differentiate between responding and non-responding patients have yet to be identified. In contrast, ketamine has the potential to elicit psychotomimetic and dissociative side effects and abuse liability, both of which could limit its use in clinical settings [24,25]. Identifying novel biomarkers capable of predicting the response to ketamine will be invaluable for selecting suitable patients for this therapy [29]. Very recently, we found that, at baseline, serum levels of IL-6 in the ketamine responder group were significantly higher than those of the control and non-responder groups [30]. In contrast, serum levels of IL-6 did not differ between control and non-responder groups. In addition, serum levels of TNF-α remained the same after ketamine infusion. These findings suggest serum IL-6 (not TNF-α) as a useful predictor for clinical outcome in patients with treatment-resistant MDD undergoing ketamine therapy [30]. The NMDA receptor antagonists such as ketamine may suppress Th1-type cytokines and shift the balance toward humoral immunity.

The current strategy for enhancing treatment outcomes in MDD relies on standardized sequential treatment algorithms and measurement-based care, but this approach is largely trial and error [2]. Incorporating reliable biomarkers into treatment algorithms could speed recovery from depression, by shortening or eliminating lengthy and ineffective clinical trials. In the future, blood levels of inflammatory molecules could serve as predictive biomarkers for antidepressant responses to several classes of antidepressants, including SSRIs, SNRIs, NRIs, NaSSAs, and NMDA receptor antagonists.
